# Fear renewal activates cyclic adenosine monophosphate signaling in the dentate gyrus

**DOI:** 10.1002/brb3.1280

**Published:** 2019-07-17

**Authors:** Yan-Wei Shi, Bu-Fang Fan, Li Xue, Xiao-Guang Wang, Xue-Ling Ou

**Affiliations:** ^1^ Faculty of Forensic Medicine Zhongshan School of Medicine, Sun Yat‐Sen University Guangzhou China; ^2^ Guangdong Province Key Laboratory of Brain Function and Disease Zhongshan School of Medicine, Sun Yat‐Sen University Guangzhou China; ^3^ Guangdong Province Translational Forensic Medicine Engineering Technology Research Center Zhongshan School of Medicine, Sun Yat‐Sen University Guangzhou China

**Keywords:** cAMP, dentate gyrus, fear conditioning, fear renewal, PDE4

## Abstract

**Background:**

Fear renewal, the context‐specific relapse of a conditioned fear after extinction, is a widely pursued model of post‐traumatic stress disorder and phobias. However, its cellular and molecular mechanisms remain poorly understood. The dentate gyrus (DG) has emerged as a critical locus of plasticity with relevance to memory, anxiety disorders, and depression, and it contributes to fear memory retrieval. Here, we have identified the role of the DG in fear renewal and its molecular mechanism.

**Materials and Methods:**

Muscimol (MUS), activator of cyclic adenosine monophosphate (cAMP) forskolin (FSK), inhibitor of protein kinase A (PKA), Rip‐cAMP, and a phosphodiesterase inhibitor rolipram were infused into DG of standard deviation rats before renewal testing. cAMP levels after fear renewal was measured by enzyme‐linked immunosorbent assay. The protein levels of phosphodiesterase 4 (PDE4) isoforms were tested by western blot. At last, the roles of cAMP signaling were also tested in the acquisition of fear conditioning, fear retrieval, and extinction.

**Results:**

Intra‐DG treatment of MUS and Rp‐cAMP impaired fear renewal. FSK and rolipram exhibited the opposite effect, which also occurred in the retrieval of original fear memory. This change in fear renewal was regulated by PDE4 isoforms PDE4A, PDE4A5, and PDE4D. In addition, FSK and rolipram facilitated the acquisition of fear conditioning in long‐term memory, but not short‐term memory, while Rp‐cAMP impaired long‐term memory. For extinction, FSK and rolipram inhibited extinction process, while Rp‐cAMP facilitated fear extinction.

**Conclusion:**

These findings demonstrated that fear renewal activated cAMP signaling in the DG through decreased PDE4 activity. Because of the role of cAMP signaling in the acquisition or retrieval of fear conditioning and encoding of extinction, it is speculated that initial learning and extinction may have similarities in molecular mechanism, especially fear retrieval and fear renewal may share cAMP signaling pathway in the DG.

## 1 INTRODUCTION

Over the last decade, extinction and fear renewal have received considerable attention with a focus on understanding their neural mechanisms. During extinction learning, there is a decrease in Pavlovian conditional responses (CRs) as a result of presenting non‐reinforced conditional stimuli (CS) (Pavlov, 1927), which models exposure therapy that is used to treat a variety of fear disorders in humans (Davis & Myers, 2002). Rather than abolish the fear memory, however, extinction learning generates a new extinction memory that competes with the fear memory for control of behavior (Bouton, 1993; Bouton & Bolles, 1979). Importantly, a fundamental observation concerning extinction is that it is context‐specific. That is, when an extinguished fear memory is encountered outside of the extinction context, renewal of the CR occurs (Bouton, 1988). Thus, renewal of fear is a major challenge for clinicians. While the neurobiology of extinction learning and retrieval has received considerable attention, the systems underlying the context dependency of extinction retrieval are not as well studied.

Fear renewal has experienced memory consolidation and may be considered as long‐term memory. The molecular mechanisms underlying long‐term consolidation mediated by cyclic adenosine monophosphate (cAMP) signaling have been extensively studied. It is thought that cAMP regulates memory formation mainly by activating the cAMP‐dependent protein kinase A (PKA). Previous studies have demonstrated that PKA may be necessary for the long‐term protein synthesis‐dependent changes that underlie L‐LTP (Arnsten, Ramos, Birnbaum, & Taylor, 2005). Inhibitors of PKA administrated into hippocampus selectively impair long‐term memory, specially the consolidation phase (Abel & Nguyen, 2008). Conversely, PKA activation in cortical regions before testing enhanced memory retrieval. Also, PKA is required for fear memory acquisition and fear extinction (Isiegas, Park, Kandel, Abel, & Lattal, 2006). Thus, cAMP/PKA signaling in cortical and hippocampal circuits plays important functions in neural plasticity, memory consolidation, and possibly the retrieval of memory.

Much researches have also focused on the major cAMP‐metabolizing enzyme phosphodiesterase 4 (PDE4) to evaluate cAMP/PKA signaling function. The PDE4 inhibitor rolipram, by enhancing cAMP signaling (Richter, Menniti, Zhang, & Conti, 2013), can shift the balance between memory extinction and strengthening the fear memory through pharmacological intervention in the dorsal hippocampus (Roesler et al., 2014). Although cAMP/PKA signaling, even PDE4, play critical roles in fear conditioning and extinction, little is known about their functions in fear renewal.

The dentate gyrus (DG) region of the hippocampal formation is considered to play a role in encoding spatial and contextual information, particularly in pattern separation and novel information (Bernier et al., 2017). A number of studies have demonstrated that suppression or lesions of the DG interferes with memory acquisition but not with the expression of previously learned memories (Kheirbek, Klemenhagen, Sahay, & Hen, 2012; Lassalle, Bataille, & Halley, 2000; Madroňal et al., 2016). However, other studies concluded that the DG contributes to both memory acquisition and retrieval (Bernier et al., 2017). These studies suggest that the contribution of the DG to stress and emotional processing, especially whether the DG participates in memory retrieval is still debated. A previous study in our laboratory demonstrated that GluR1‐ser845 phosphorylated by PKA in hippocampal CA1 increased after fear renewal (Xue et al., 2014). Furthermore, DG inhibition could cause a rapid loss of Schaffer collateral (SC) synaptic plasticity linking CA3 and CA1 and conditioned responding to CS (Madroňal et al., 2016). So it is speculated that cAMP/PKA signaling in the DG may play important functions in fear renewal. The present study especially focused on the DG as an important brain region involved in fear renewal and tested whether cAMP signaling is necessary for fear renewal.

## 2 MATERIALS AND METHODS

### 2.1 Experiment 1

#### 2.1.1 Subjects

Adult male Sprague‐Dawley rats (220–250 g) obtained from the Zhongshan School of Medicine at Sun Yat‐Sen University were individually housed on a 12/12 hr light/dark cycle (lights on at 6:00 a.m.) in Plexiglas cages and had access to food and water ad libitum. Food and water were supplied throughout the duration of the experiments. Rats were handled for 7 days before the experiment. All procedures were approved by the Institutional Animal Care and Use Committee of the Zhongshan School of Medicine at Sun Yat‐Sen University and complied with the National Institutes of Health Guide for the Care and Use of Laboratory Animals.

#### 2.1.2 Apparatus

Behavioral experiments were conducted in observation chambers (30 × 24 × 21 cm; Coulbourn Instruments, Lehigh Valley, PA) constructed from aluminum and Plexiglas. Each chamber was situated in a sound‐attenuating cabinet located in a brightly lit and isolated room. The floor of each chamber consisted of 19 stainless steel rods (4 mm in diameter) was spaced 1.5 cm apart (center to center). Foot shocks produced by foot rods wired to a shock source were used as unconditional stimuli (US). In addition, the acoustic CS was delivered by the speaker in one wall of the chamber. Above each chamber, a closed‐circuit video camera was used to record the behavior of each rat. Sensory stimuli were adjusted within the chambers to generate three distinct contexts (A, B, and C) that differed in transported boxes, illumination of the house and chambers, background noises, chamber cleaner, etc. For context A, a 40‐W red house light mounted opposite to the speaker was turned on, and the room light remained off. Ventilation fans were turned off and the chamber was cleaned with 70% ethanol. Rats were transported to context A in white plastic boxes without bedding. For context B, house lights were turned off and the overhead lighting was a 15‐W white light. Ventilation fans were turned on, each sidewall of the chamber was covered with white paper, and the chambers were cleaned with 1% acetic acid. Rats were transported to context B in black plastic boxes without bedding. For context C, both the 40‐W house light and the 15‐W overhead light were turned on. Ventilation fans were off, each sidewall of the chamber was covered with red paper, and the chambers were cleaned with 1% ammonium hydroxide. Rats were transported to context C in white cultivation cans with bedding. In each context, stainless steel pans were filled with a thin layer of the respective odors of the contexts and inserted below the grid floor. Sensory stimuli, senses of sight, hearing, smell, and touch, were adjusted within the chambers to generate maximum distinct contexts. The different contexts were counterbalanced for conditioning, extinction, and testing (Jin & Maren, 2015; Xue et al., 2014).

#### 2.1.3 Surgery

Rats were anesthetized with sodium pentobarbital (65 mg/kg, i.p.) (Corcoran & Maren, 2001; Hobin, Ji, & Maren, 2006) and were subsequently secured in a stereotaxic apparatus (RWD Life Science, Shenzhen, China). Infusion cannulae were replaced with dummy cannulae that were cut to extend 0.5 mm beyond the guide cannulae to prevent clogging. Stereotaxic coordinates were determined according to the Paxinos and Watson (2009) and Kesner, Kirk, Yu, Polansky, and Musso (2016); DG: 2.7 mm posterior to bregma, 2.1 mm lateral to midline, 3.4 mm ventral from dura. A 28‐gauge dummy cannula was inserted into each cannula to prevent clogging. Three jewelry screws were implanted over the skull to serve as anchors, and the whole assembly was affixed to the skull with dental cement. The surgery was finished by the different authors to confirm cannula placement. The rats were monitored and handled daily and were given 7 days to recover from surgery. All surgical procedures were conducted in accordance to the National Institutes of Health Guide for the Care and Use of Laboratory Animals.

#### 2.1.4 Drugs

Muscimol (MUS), GABA_A_ agonist, was used to inactive DG, and was infused 20–25 min before the retrieval test. Obturators were removed and injectors were placed into the guide cannulas, and then rats received an infusion of 0.9% sterile saline (SAL group) or (MUS group; 1 μg/μl dissolved in 0.9% sterile saline) at a rate of 0.25 μL/min for 0.5 μl per side (Barbosa, Pontes, Ribeiro, Ribeiro, & Silva, 2012; Fu et al., 2016). After infusion, the cannulae were left in place for 2 min to allow diffusion of the drug from the tip.

#### 2.1.5 Behavioral procedures

Rats were subjected to four experimental phases: acclimation, fear conditioning, extinction, and retrieval test. On the first day, rats were preexposed to 5 tones (30 s, 4 kHz, 75 dB) in context A. On the second day, rats were placed in the conditioning chamber (context A) and received three CS tones co‐terminated with foot shock (1 s, 0.6 mA) trials (2–4‐min inter‐trial intervals [ITIs], average ITI: 3 min) beginning 3 min after being placed in context A. Then, 60 s after the final shock, the rats were returned to their home cages. Twenty‐four hours after the conditioning session, the extinction training, which included 40 CS‐only presentations, was performed in the chamber with context B. During this period, rats assigned to the experimental or control group were presented with 40 tones (30 s, 75 dB, 4 kHz; average ITI: 1.5 min) without a foot shock. Rats that showed less than 50% freezing during the first 5 tones were excluded from the subsequent study phases (Yang, Chao, & Lu, 2006). On the last day at 24 hr after extinction, the rats tested in context C were classified as the ABC group. The rats tested in context B were classified as the ABB group. Then according to the infused drug, four groups were set up in this experiment: SAL‐ABB group, SAL‐ABC group, MUS‐ABB group, and MUS‐ABC group (*n* = 8 in each group). The behavioral experiment was blinded to drug treatment.

#### 2.1.6 Data collection and statistical analysis

Freezing was used to measure conditioned fear and was continuously recorded during the conditioning session and later scored to determine the degree to which the rats acquired the conditional association. Behavioral data were recorded with digital video cameras automatically, and freezing was quantified from digitized video images using FreezeView2 software. All data are expressed as the mean ± standard deviation (*SD*). Statistical analysis blinded to data collecting was performed using unpaired *t* test and analysis of variance (ANOVA) with Bonferroni post hoc comparisons, which were performed after a significant overall *F* ratio.

#### 2.1.7 Histology

**Figure 1:**
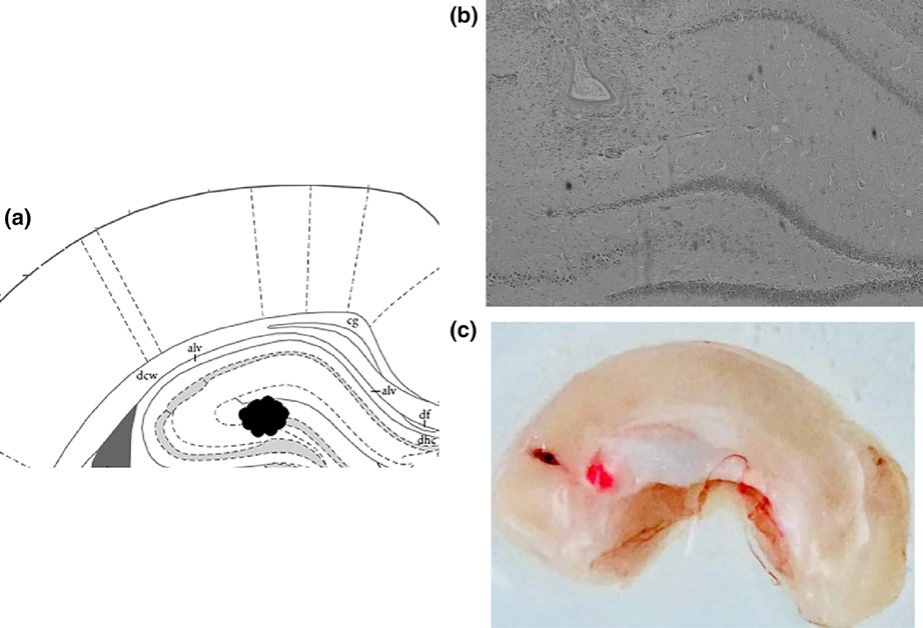
Cannula tip placement for animals included in the analysis. (a and b) The diagram shows a coronal view of rat brain at a position 2.76 mm posterior to bregma and the injection sites are indicated by black dots. (c) The same volume of stain solution as all the drugs in this study were infused into this site and did not spread to other areas

Following the retrieval test, the animals were given an overdose of sodium pentobarbital and microinjected with methylene blue (1%, 1 µl) to mark the drug infusion site. Then, the brains were removed. Sections were examined to determine the locations of the cannulae aimed toward the DG. The cannula placements were verified using a rat brain atlas. Only rats with cannula tips at or within the boundaries of the DG were included in the data analyses (Figure 1). There were nine rats excluded due to off‐target cannula.

### 2.2 Experiment 2

#### 2.2.1 Subjects

The subjects were Sprague‐Dawley rats (220–250 g), obtained and housed as described in Experiment 1.

#### 2.2.2 Apparatus

Behavioral apparatus and the context design were as described in Experiment 1.

#### 2.2.3 Behavioral procedures

The rats were divided into four groups: the naive group (*n* = 4), the no‐extinction (NE) group (*n* = 5), the ABB group (*n* = 4), and the ABC group (*n* = 4), The ABB group and the ABC group were set up as the procedures described in Experiment 1. The naive group was handled and exposed to the conditioning box for an equivalent amount of time but were not exposed to tones or shocks. The NE group did not experience extinction and tested in context B at the same time as the ABC and the ABB group. At 1 hr after the retrieval test, rats were deeply anesthetized with pentobarbital (Nembutal; 65 mg/kg, i.p.) and decapitated. The DG was dissected and stored at −80°C or in liquid nitrogen until processed.

#### 2.2.4 cAMP immunoassay

The frozen DG tissue samples were recovered to room temperature, 20 μl of 0.01 mM modified PBS (MPBS) buffer was added per mg tissue, samples were homogenized using an ultrasonic homogenizer, and homogenates were centrifuged for 20 min at 10,000 *g* at 4°C. cAMP protein was quantified using an enzyme‐linked immunosorbent assay kit (cAMP Enzyme Immunoassay Kit, Direct, Sigma‐Aldrich). The sample handling and quantitative methods were performed according to the manufacturer's instructions. Total protein concentration was determined using a Bradford method protein assay (Bio‐Rad, CA).

#### 2.2.5 Data collection and statistical analysis

Behavioral data collection and analysis were as described in Experiment 1. The data of cAMP protein concentration were performed using ANOVA with Bonferroni post hoc comparisons, which were performed after a significant overall *F* ratio. Correlation in freezing levels with cAMP and proteins levels were analyzed by spearman's correlation.

### 2.3 Experiment 3

#### 2.3.1 Subjects

The subjects were Sprague‐Dawley rats (220–250 g), obtained and housed as described in Experiment 1.

#### 2.3.2 Surgery

Surgery methods were as described in Experiment 1.

#### 2.3.3 Drugs

The adenylate cyclase activator forskolin (FSK [*n* = 8]; 1 mM, 0.25 μl per side) was used to activate the cAMP pathway specifically, or vehicle (5% vol/vol dimethyl sulfoxide [DMSO] [*n* = 8] in saline; Ghosh & Chattarji, 2015) was injected. The PKA inhibitor Rp‐cAMP (0.5 μg in 0.5 μl per side, *n* = 8), or vehicle (10% DMSO in saline, *n* = 8) was infused (Moncada, Ballarini, Martinez, Frey, & Viola, 2011). All drugs were purchased from Sigma‐Aldrich Co. and were infused 30 min before the retrieval test on the day 4 at a rate of 0.25 μl/min. Then all rats were tested in context C.

#### 2.3.4 Behavioral procedures and apparatus

Only the ABC group was used in this experiment. Behavioral procedures and apparatus were as described in Experiment 1.

#### 2.3.5 Data collection and statistical analysis

Trails were averaged in blocks of four during extinction. Unpaired *t* test was used to analyze the behavioral data aimed to assess the role of drugs in the retrieval test. Repeated measures ANOVA was used to analyze the effect of drugs and trials during extinction. Data are represented as means ± *SD*.

### 2.4 Experiment 4

#### 2.4.1 Subjects

The subjects were Sprague‐Dawley rats (220–250 g), obtained and housed as described in Experiment 1.

#### 2.4.2 Surgery

Surgery methods were as described in Experiment 1.

#### 2.4.3 Drugs

To pharmacologically inhibit PDE4, the animals received a bilateral 0.5‐μl infusion of the PDE4‐selective inhibitor rolipram (7.5 μg/side dissolved in vehicle) (*n* = 11), or vehicle (20% DMSO in saline) (*n* = 11) was administered (Werenicz et al., 2012). The drugs were purchased from Sigma‐Aldrich Co. and were infused 30 min before the retrieval test on the day 4 at a rate of 0.25 μl/min. Then all rats were tested in context C.

#### 2.4.4 Behavioral procedures and apparatus

Only the ABC group was used in behavior pharmacological experiment. Behavioral procedures and apparatus were as described in Experiment 1.

In the experiment of accessing protein level, rats without any surgery were divided into four groups randomly: the naïve group, the NE group, the ABB group, and the ABC group (*n* = 5 in each group). Behavioral procedures and apparatus were as described in Experiment 22.1.4.

#### 2.4.5 Western blotting

After behavioral testing, animals were anesthetized with sodium pentobarbital and decapitated. Then, coronal brain slices (400 µm thick) containing the amygdala were prepared. Tissue blocks from the DG were obtained from three consecutive 400‐µm sections with the aid of a microscope, and approximately 80% of the identifying regions were included to reduce contamination by other tissues. The DG samples were dissected as quickly as possible from the coronal slices, placed on ice under a dissecting microscope, and preserved in liquid nitrogen to avoid dephosphorylation and protein degradation. Samples were ground with a high‐flux tissue grinder for 90 s. The supernatant was then assayed for total protein concentration using a bicinchoninic acid (BCA) protein assay kit.

Western blotting was performed using a Wes Simple Western system, an automated capillary‐based size‐sorting system (ProteinSimple, San Jose, CA). All procedures were performed using the manufacturer's reagents according to their user manual. Briefly, 8 μl of diluted protein lysate was mixed with 2 μl of 5× fluorescent master mix and heated at 95°C for 5 min. The samples (1 μg), blocking reagent, wash buffer, primary antibodies, secondary antibodies, and chemiluminescent substrate were dispensed into the designated wells of a manufacturer‐provided microplate. The plate was loaded into the instrument, and protein was drawn into individual capillaries on a 25‐capillary cassette provided by the manufacturer. Protein separation and immunodetection were performed automatically on the individual capillaries using default settings. The data were analyzed using Compass software (ProteinSimple) and produced digital bands that match the data. The primary antibodies were anti‐PDE4A (Abcam), anti‐PDE4A5 (Abcam), anti‐PDE4B (Abcam), and anti‐PDE4D (Abcam); and β‐actin was used as a loading control (rabbit).

#### 2.4.6 Data collection and statistical analysis

Analyzing the behavioral data was described in Experiment 32.2.2. The data of protein concentration were performed using ANOVA with Bonferroni post hoc comparisons.

### 2.5 Experiment 5

#### 2.5.1 Subjects

The subjects were Sprague‐Dawley rats (220–250 g), obtained and housed as described in Experiment 1.

#### 2.5.2 Surgery

Surgery methods were as described in Experiment 1.

#### 2.5.3 Drugs

Forsklin, Rp‐cAMP, rolipram, and their control group respectively were as described in Experiment 3 (*n* = 10 in each group). All the drugs were administrated into the DG 30 min before fear memory retrieval at a rate of 0.25 μl/min only in the NE group.

#### 2.5.4 Behavioral procedures and apparatus

Only the NE group was used in this experiment. Behavioral procedures and apparatus were as described in Experiment 1.

#### 2.5.5 Data collection and statistical analysis

Data collection was as described in Experiment 1. All data are expressed as the mean ± *SD*. Statistical analysis was performed using unpaired *t* test.

### 2.6 Experiment 6

#### 2.6.1 Subjects

The subjects were Sprague‐Dawley rats (220–250 g), obtained and housed as described in Experiment 1.

#### 2.6.2 Surgery

Surgery methods were as described in Experiment 1.

#### 2.6.3 Drugs

Forsklin (*n* = 6), Rp‐cAMP (*n* = 6), rolipram (*n* = 8), and their control groups (5% DMSO: *n* = 7; 10% DMSO: *n* = 7; 20% DMSO: *n* = 8) respectively were as described in Experiment 3. All the drugs were administrated into the DG 30 min before fear conditioning acquisition at a rate of 0.25 μl/min.

#### 2.6.4 Behavioral procedures and apparatus

Fear conditioning behavioral procedures and apparatus were as described in Experiment 1. Short‐term memory was tested 1 hr after fear conditioning and long‐term‐memory was tested 24 hr after conditioning.

#### 2.6.5 Data collection and statistical analysis

Data collection was as described in Experiment 1. All data are expressed as the mean ± *SD*. Repeated measures ANOVA was used to analyze the effect of drugs and trials during fear conditioning. The data of short‐term memory and long‐term memory were performed using unpaired *t* test.

### 2.7 Experiment 7

#### 2.7.1 Subjects

The subjects were Sprague‐Dawley rats (220–250 g), obtained and housed as described in Experiment 1.

#### 2.7.2 Surgery

Surgery methods were as described in Experiment 1.

#### 2.7.3 Drugs

Forsklin (*n* = 8), Rp‐cAMP (*n* = 8), rolipram (*n* = 8), and their control groups (5% DMSO: *n* = 8; 10% DMSO: *n* = 7; 20% DMSO: *n* = 8), respectively were as described in Experiment 32.2.2. All the drugs were administrated into the DG 30 min before extinction at a rate of 0.25 μl/min.

#### 2.7.4 Behavioral procedures and apparatus

Extinction behavioral procedures and apparatus were as described in Experiment 1.

#### 2.7.5 Data collection and statistical analysis

Data collection was as described in Experiment 1. All data are expressed as the mean ± *SD*. Repeated measures ANOVA was used to analyze the effect of drugs and trials during extinction.

## 3 RESULTS

### 3.1 Inactivation of the DG impairs fear renewal

**Figure 2:**
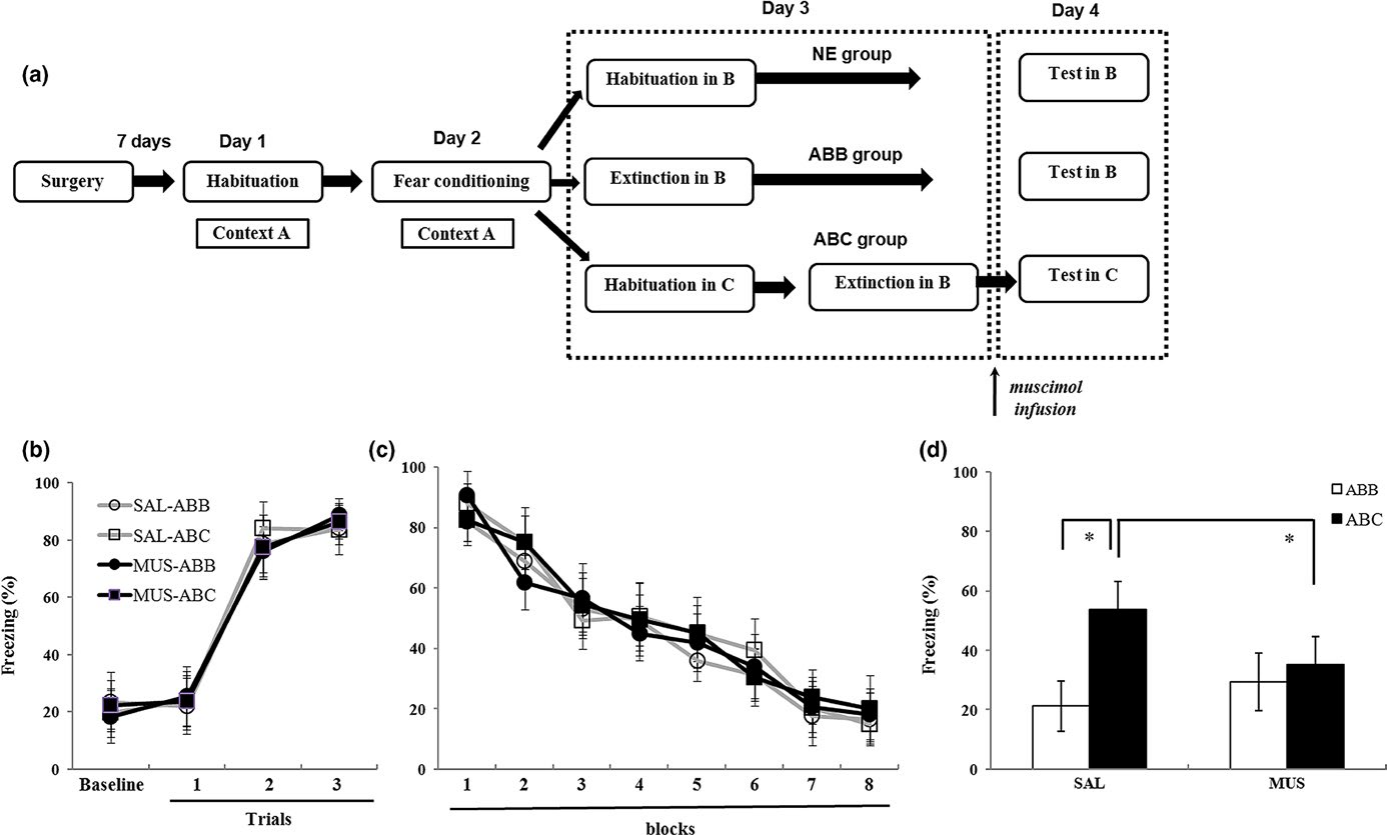
DG inactivation before the retrieval test impairs fear renewal. (a) An outline of the procedure for the MUS administration experiment. (b) Mean (±*SD*) percentage of freezing during fear conditioning. Freezing was averaged over a 3‐min baseline without any tone or shock. Each trial consisted of average freezing during each CS presentation and the subsequent ITI. (c) Mean (±*SD*) percentage of freezing during the 40 tone‐only extinction session. Data are presented as 8 five‐trial blocks. Each trial consisted of average freezing during each CS presentation and the subsequent ITI. (d) Freezing percentage (mean ± *SD*) across the 5 tones of the testing session. Rats were tested in a context that was either consistent (open bars) or inconsistent (filled bars) with context A (*p < 0.05, *n* = 8 in each group). DG, dentate gyrus; *SD*, standard deviation; MUS, Muscimol; CS, conditional stimuli; ITI, inter‐trial interval

In the first experiment, the role of the DG in extinction memory retrieval was evaluated. The experiment protocol is shown in Figure 2a. All rats showed significantly increased freezing time during the conditioning session (effect of block, *F*_(3,93)_ = 499.7, *p* < 0.001). The *df* in *F*_(3,93) _was calculated that “3 = 4 blocks‐1; 93 = (32 rats − 1) × (4 − 1),” and neither the percentage of freezing nor the interaction differed between the groups (*F*s < 1; Figure 2b). For the extinction sessions, conditioned freezing declined significantly across the extinction [effect of block, *F*_(7,217)_ = 187.7, *p* < 0.001; The *df* in *F*_(7,217)_ was calculated that “7 = 8 blocks‐1; 217 = (32 rats − 1) × (8 − 1)”Figure 2c]. After extinction, rats were infused with SAL or with MUS to inactivate the DG 20 min prior to retrieval test either in the extinction context (designated as the ABB group) or in a novel context (designated as the ABC group). There was significant interaction between the drug and context by two‐way ANOVA (*F*_(3,28)_ = 16.75, *p* < 0.001. The *df* in *F*_(3,28)_ was calculated that “3 = 4 groups − 1; 28 = 32 rats – 4.” As shown in Figure 2d, the SAL‐ABC group exhibited a robust renewal of fear to the extinguished CS when tested in the novel context (*t*_(1.14)_ = −6.86, *p* < 0.001). However, this effect was eliminated in rats in the MUS‐ABC group (*t*_(1,14)_ = −1.24, *p* = 0.23). Bonferroni post hoc comparisons demonstrated that there were significant differences between SAL‐ABC and MUS‐ABC (*p* = 0.004). These results suggested that the DG is a critical hub for fear renewal.

### 3.2 Fear renewal increases cAMP levels in the DG

**Figure 3:**
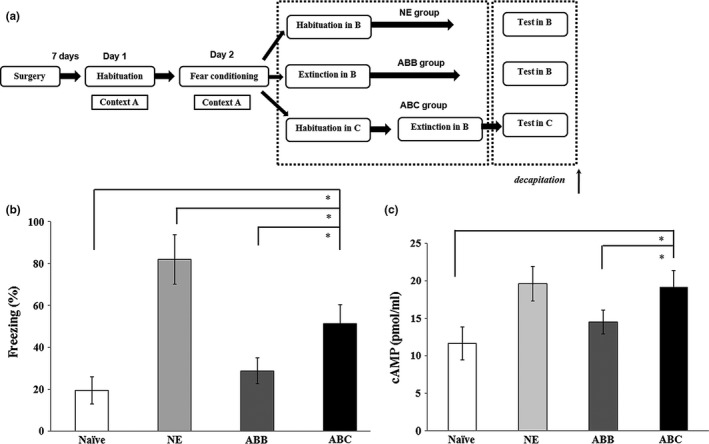
Fear renewal increases cAMP levels in the DG. (a) An outline of the procedure for rat decapitation. (b) Rats in these four groups were given 5 tones of the testing prior to decapitation (*p < 0.05, *n* = 4, 5, 4, 4 respectively in each group). (c) Fear renewal increased cAMP protein levels in the DG (*p < 0.05, *n* = 4, 5, 4, 4 respectively in each group). cAMP, cyclic adenosine monophosphate; DG, dentate gyrus

To determine the molecular mechanisms by which the DG is involved in fear renewal, the cAMP levels induced by fear renewal were detected using biochemical assays in four groups: the naïve group of rats without any behavioral training or drug administration, the NE group of rats that underwent fear conditioning without extinction, the ABB group, and the ABC group. Ten rats in each group were used, of which five rats were tested for cAMP levels and others were used to investigate isoforms of PDE4 protein levels. These four groups were all tested prior to decapitation. A one‐way ANOVA revealed that there were significant differences among the groups (*F*_(3,13)_ = 13.11, *p* < 0.05). Post hoc comparisons revealed that the NE group freezing levels were significantly higher than the ABB group, which is extinguished, and the naïve group, but not significant different to the ABC group (*p* > 0.05). And in the ABB group, the freezing levels were significantly lower than the ABC group (*p* < 0.05) (Figure 3a). In addition, the correlations between cAMP levels (Figure 3b) and the freezing levels (Figure 3a) were significant (*p* < 0.001).

As to cAMP levels, there were significant differences among the groups (*F*_(3,13)_ = 15.07, *p* < 0.05). Post hoc comparisons revealed that cAMP levels in the ABC group were significantly higher than those in the ABB group (*p* < 0.05) and the naïve group (*p* < 0.05), while there were no significant differences in cAMP levels between the NE group and the ABC group (*p* > 0.05; Figure 3b). The results demonstrated that fear can increase the cAMP levels in the DG.

### 3.3 Activation or inhibition of cAMP signaling in the DG facilitates or impairs fear renewal

**Figure 4:**
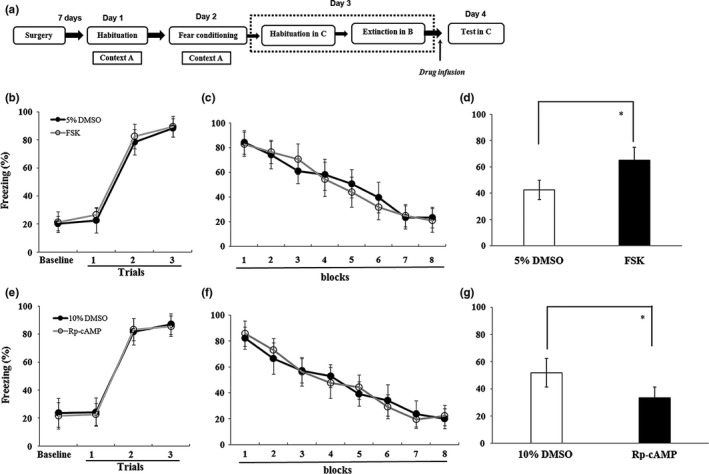
Effects of the cAMP activator FSK and the PKA inhibitor Rp‐cAMP on fear renewal when rats were tested in context C, which was inconsistent with the fear conditioning training in context A. (a) An outline of the procedure for the FSK and Rp‐cAMP administration experiments. (b and e) Mean (±*SD*) percentage of freezing during fear conditioning. Freezing was averaged over a 3‐min CS without any tone or shock. Each trial consisted of average freezing during each CS presentation and the subsequent ITI. (c and f) Mean (±*SD*) percentage of freezing during the 40 tone‐only extinction session. Data are presented as 8 five‐trial blocks. Each trial consisted of average freezing during each CS presentation and the subsequent ITI. (d) FSK increased fear renewal significantly across the 5 tones of the testing session. (g) Rp‐cAMP decreased fear renewal significantly across the 5 tones of the testing session (*p < 0.05, *n* = 11 in each group). cAMP, cyclic adenosine monophosphate; CS, conditional stimuli; FSK, forskolin; ITI, inter‐trial interval; PKA, protein kinase A; *SD*, standard deviation

To explore whether cAMP level changes in the DG might be related to fear renewal, a bilateral intra‐DG infusion of the adenylate cyclase activator FSK was administered to increase cAMP levels (Vecsey et al., 2009). DMSO (5%) was used as a control (Figure 4a). All rats increased their freezing behavior during fear conditioning (main effect of block, *F*_(3,84)_ = 482.02, *p* < 0.001), and the percentage of freezing time did not differ between the groups (main effect of group and group × block interaction, *F*s < 1; Figure 4b). During the extinction session, rats exhibited a similar reduction in freezing across the extinction session (main effect of block, *F*_(7,168)_=97.77, *p* < 0.001; main effect of group and group × block interaction, *F*s < 1.4; Figure 4c). In the FSK experiment, the percentage of freezing time in the FSK group was significantly higher than that in the control group (*t*_(1,20)_ = −4.55, *p* < 0.05; Figure 4d).

cAMP‐dependent PKA is the specific target of cAMP, and its activity is stimulated by cAMP. Thus, Rp‐cAMP, a competitive inhibitor and antagonist of PKA, was used to indirectly inhibit cAMP activity. As in the previous FSK experiment, all rats increased their conditioned freezing behavior (main effect of block, *F*_(3,84)_ = 356.32, *p* < 0.001), and the percentage of freezing did not differ between the groups (main effect of group and group × block interaction, *F*s < 1; Figure 4e). During the extinction session, rats exhibited a similar reduction in the percentage of freezing across the extinction session (main effect of block, *F*_(7,168)_ = 119.4, *p* < 0.001; main effect of group and group × block interaction, *F*s < 1.4; Figure 4f). Before the rats were tested in context C (the ABC group), they were infused with Rp‐cAMP or 10% DMSO as a control. The percentage of freezing time in the Rp‐cAMP group was significantly lower than that in the control group (*t*_(1,20)_ = 4.44, *p* < 0.05; Figure 4g). These data revealed that disruption or activation of cAMP signaling could impair or facilitate fear renewal, respectively.

### 3.4 Fear renewal reduces PDE4 isoform protein levels in the DG

**Figure 5:**
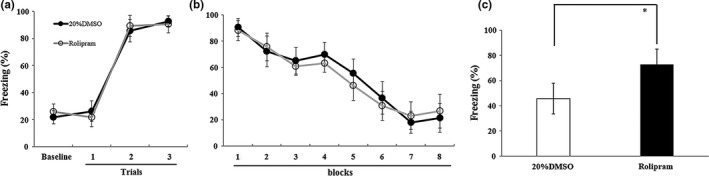
Effect of the PDE4‐selective inhibitor rolipram on fear renewal when rats were tested in context C, which was inconsistent with the fear conditioning training in context A. (a) Mean (±*SD*) percentage of freezing during fear conditioning. Freezing was averaged over a 3‐min baseline without any tone or shock. Each trial consisted of average freezing during each CS presentation and the subsequent ITI. (b) Mean (±*SD*) percentage of freezing during the 40 tone‐only extinction session. Data are presented as 8 five‐trial blocks. Each trial consisted of average freezing during each CS presentation and the subsequent ITI. (c) Freezing across the 5 tones of the testing session (*p < 0.05, *n* = 10 in each group). CS, conditional stimuli; ITI, inter‐trial interval; PDE4, phosphodiesterase 4; *SD*, standard deviation

The cAMP‐specific PDE4 family plays a major role in regulating cAMP signaling in the brain (Vecsey et al., 2009). Therefore, we tested whether fear renewal affects PDE4 activity or levels in the DG. The procedures for this experiment was similar to those in Section 3.32.7.5 except that all rats were conditioned, extinguished, and tested using the ABC procedure. All rats increased their conditioned freezing (main effect of block, *F*_(3,36)_ = 679.18, *p* < 0.001), and the percentage of freezing time did not differ between the groups (main effect of group and group × block interaction, *F*s < 1; Figure 5a). During the extinction session, rats exhibited a similar reduction in the percentage of freezing time across the extinction session (main effect of block, *F*_(7,152)_ = 127.08, *p* < 0.001; main effect of group and group × block interaction, *F*s < 1.9; Figure 5b). First, the PDE4‐selective inhibitor rolipram was infused into the DG before the retrieval test to determine whether PDE4 activity is involved in fear renewal. Rats administered with rolipram showed a significantly higher percentage of freezing time than the SAL group (*t*_(1,18)_ = −5.00, *p* < 0.05; Figure 5c).

**Figure 6:**
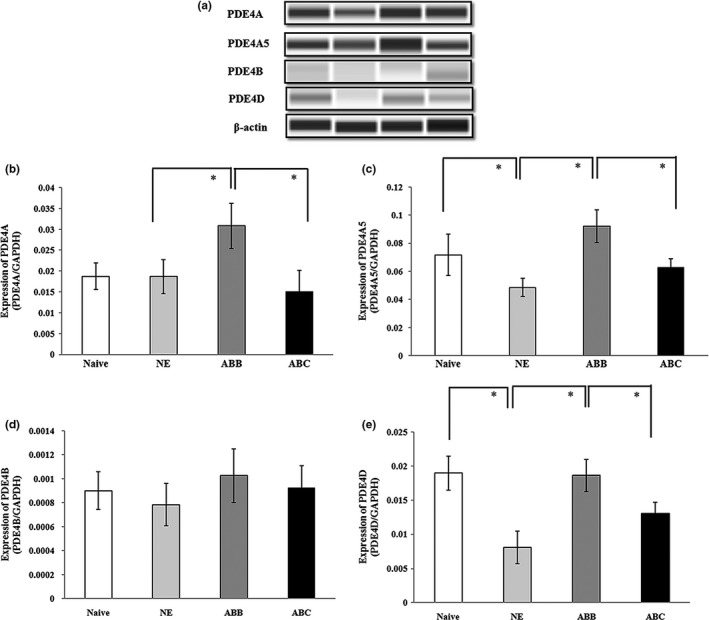
Protein expression of PDE4 isoforms induced by fear renewal. (a) The representative blots of PDE4A5, PDE4A, PDE4B and PDE4D from rats decapitated after extinction testing. (b–e) Mean (±*SD*) of PDE4A5, PDE4A, PDE4B, and PDE4D protein levels (*p < 0.05, *n* = 5 in each group). PDE4, phosphodiesterase 4; *SD*, standard deviation

Next, we assessed the protein levels of the PDE4 isoforms (PDE4A, PDE4A5, PDE4B, and PDE4D) in the DG in four groups: the naïve group, the NE group, the ABB group, and the ABC group. For PDE4A, there were significant differences among the groups (*F*_(3,16)_ = 11.71, *p* < 0.05). Post hoc comparisons revealed that the protein levels in the ABB group were greatly increased compared to the NE group (*p* < 0.05) and the ABC group (*p* < 0.05). However, there were no significant differences among the naïve group, the NE group and the ABC group (*p* > 0.05; Figure 6a,b). For PDE4A5, there were significant differences among the groups (*F*_(3,16)_ = 15.93, *p* < 0.05). Post hoc comparisons revealed that the expression in the ABC group was significantly reduced compared to the ABB group (*p* < 0.05). In addition, the protein level in the NE group was significantly less than that in the naïve group (*p* < 0.05) and the ABB group (*p* < 0.05). There were no significant differences between the ABC group and the NE group (*p* > 0.05; Figure 6a,c). We found that the expression of PDE4B showed no significant differences among all groups (*F*_(3,16)_ = 1.40, *p* > 0.05; Figure 6a,d). For PDE4D, there were significant differences among the groups (*F*_(3,16)_ = 25.71, *p* < 0.05). The protein levels of PDE4D in the ABB group were apparently higher than those in the NE group (*p* < 0.05) and significantly lower than the ABC group (*p* < 0.05) and the naïve group (*p* < 0.05; Figure 6a,e). PDE4D levels in the naïve group were significantly higher than NE and the ABC group (*p* < 0.05). These findings demonstrated that fear renewal could regulate the protein levels of PDE4A, PDE4A5, and PDE4D in the DG. In addition, the correlations between the three protein levels and the freezing levels (Figure 3a) were all significant (*p* < 0.05).

### 3.5 Involvement of cAMP signaling in the expression of fear conditioning

**Figure 7:**
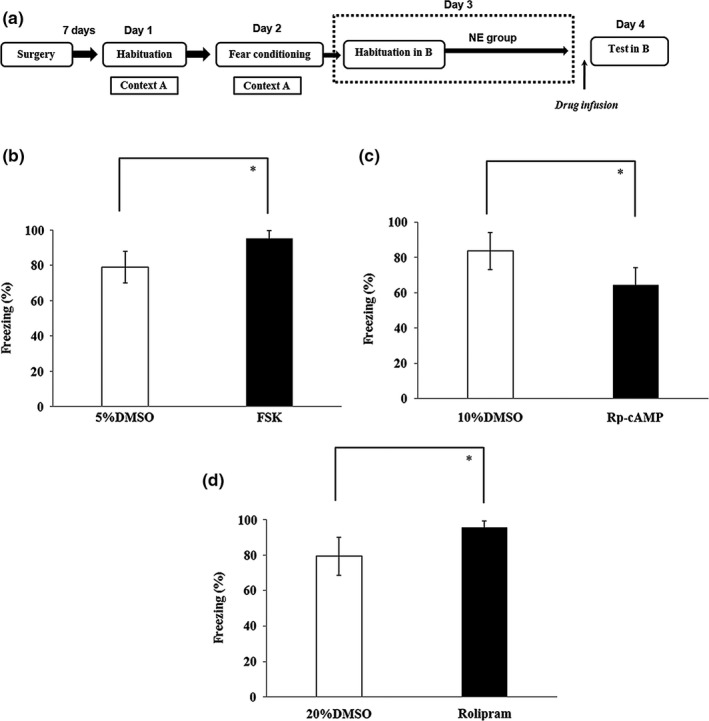
cAMP signaling has the same effect with the fear memory retrieval in the NE group. (a) An outline of the procedure for the FSK, Rp‐cAMP and rolipram administration experiments. (b) Intra‐DG infusion of FSK could facilitate fear memory retrieval. (c) Intra‐DG infusion of Rp‐cAMP inhibited fear memory retrieval. (d) Intra‐DG infusion of rolipram could facilitate fear memory retrieval (*p < 0.05, *n* = 10 in each group). cAMP, cyclic adenosine monophosphate; NE, no‐extinction; FSK, forskolin; DG, dentate gyrus

To test whether inhibition of cAMP signaling in the DG is specific to renewal, fosklin, Rp‐cAMP, and rolipram were injected into the DG before fear memory retrieval in NE group (Figure 7a). The control NE group was significantly different from FSK group (*t*_(1,18)_ = −5.04, *p* < 0.05; Figure 7b), Rp‐cAMP group (*t*_(1,18)_ = 4.22, *p* < 0.05; Figure 7c), and rolipram group (*t*_(1,18)_ = −4.60, *p* < 0.05; Figure 7d). These results suggested that cAMP signaling also effects the retrieval of original fear memory.

### 3.6 Involvement of cAMP signaling in the acquisition of fear conditioning

**Figure 8:**
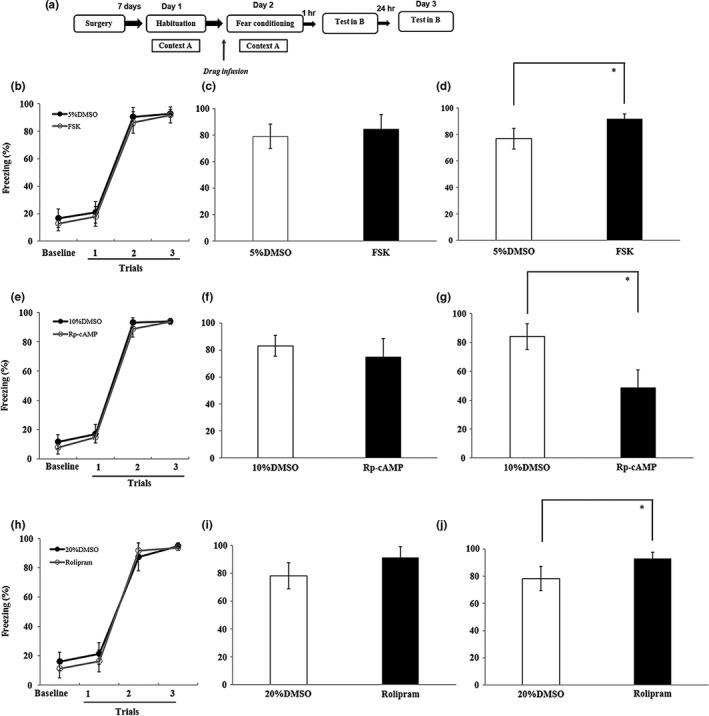
Effect of cAMP signaling in acquisition of fear conditioning. (a) An outline of the procedure for the FSK, Rp‐cAMP, and rolipram administration experiments. (b–d) Intra‐DG infusion of FSK before fear conditioning could facilitate long‐term memory (*n* = 7 in 5% DMSO group; *n* = 6 in FSK group). (e–g) Intra‐DG infusion of Rp‐cAMP before fear conditioning could impair long‐term memory (*n* = 7 in 10% DMSO group; *n* = 7 in Rp‐cAMP group). (h–j) Intra‐DG infusion of rolipram before fear conditioning could facilitate long‐term memory (*n* = 8 in 20% DMSO group; *n* = 8 in rolipram group) (*p < 0.05). cAMP, cyclic adenosine monophosphate; FSK, forskolin; DG, dentate gyrus; DMSO, dimethyl sulfoxide

Drugs were infused into the DG before fear conditioning (Figure 8a). The percentage of freezing time during the encoding of fear conditioning did not differ between the FSK groups and the 5% DMSO group (*F*_(1,11)_ = 2.24, *p* = 0.142, Figure 8b). Although there were no significant effects on the short‐term memory (*t*_(1,11)_ = −0.88, *p* = 0.4, Figure 8c), the long‐term memory significantly increased (*t*_(1,11)_ = −4.34, *p* = 0.001, Figure 8d).

For Rp‐cAMP treated experiment, ANOVA revealed no main effect of drug treated (*F*_(1,11)_ = 4.9, *p* = 0.061, Figure 8e) during the encoding of fear conditioning and the short‐term memory (*t*_(1,11)_ = 1.37, *p* = 0.199, Figure 8f). Also, Rp‐cAMP decreased significantly in the long‐term memory (*t*_(1,11)_ = 6.023, *p* < 0.05, Figure 8g).

For rolipram‐treated experiment, there were also no significant differences during fear conditioning (*F*_(1,14)_ = 0.44, *p* = 0.51, Figure 8h). And rolipram‐treated rats showed lower levels of freezing compared with control group in the long‐term memory (*t*_(1,14)_ = −3.991, *p* < 0.05, Figure 8j), but without significant differences in short‐term memory (*t*_(1,14)_ = 1.345, *p* = 0.202, Figure 8i).

### 3.7 Involvement of cAMP signaling in extinction

**Figure 9:**
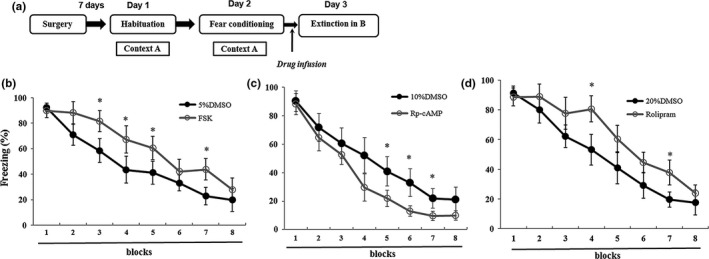
Effect of cAMP signaling in extinction. (a) An outline of the procedure for the FSK, Rp‐cAMP and rolipram administration experiments. (b) Intra‐DG infusion of FSK before extinction could impair extinction process (*n* = 8 in each group). (c) Intra‐DG infusion of Rp‐cAMP before extinction could facilitate extinction process (*n* = 7 in 10% DMSO group; *n* = 8 in Rp‐cAMP group). (d) Intra‐DG infusion of rolipram before extinction could impair extinction process (*n* = 8 in each group) (*p < 0.05). cAMP, cyclic adenosine monophosphate; FSK, forskolin; DG, dentate gyrus; DMSO, dimethyl sulfoxide

To test whether cAMP signaling has effect on extinction, FSK, Rp‐cAMP, and rolipram were infused into the DG before extinction (Figure 9a). For FSK‐treated experiment, FSK‐treated rats showed lower levels of freezing compared with the control group during extinction (*F*_(1,14)_ = 90.96, *p* < 0.05), especially in block 3 (*t*_(1,14)_ = −5.33, *p* < 0.05), 4 (*t*_(1,14)_ = −4.45, *p* = 0.001), 5 (*t*_(1,14)_ = 4.26, *p* = 0.001) and 7 (*t*_(1,14)_ = −5.50, *p* < 0.05) (Figure 9b).

For Rp‐cAMP treated experiment, Rp‐cAMP treated rats' significant differences compared with the control group during the final three blocks (*F*_(1,13)_ = 49.52, *p* < 0.05; block 4: *t*_(1,13)_ = 2.99, *p* = 0.001; block 5: *t*_(1,13)_ = 2.38, *p* = 0.032; block 6: *t*_(1,13)_ = 2.37, *p* = 0.033). It is suggested that Rp‐cAMP could facilitate extinction (Figure 9c).

For rolipram‐treated experiment, there were significant differences between rolipram‐rats and the control group during extinction (*F*_(1,14)_ = 7.29, *p* < 0.05). Also, freezing levels were increased significantly in block 4 (*t*_(1,14)_ = −5.75, *p* < 0.05) and 7 (*t*_(1,14)_ = −4.02, *p* < 0.05). It is suggested that rolipram could inhibit extinction (Figure 9d).

## 4 DISCUSSION

The results presented in this study provide the evidence of cAMP signaling required for activation of the DG for fear renewal. First, infusion of the GABA_A_ agonist MUS into the DG before extinction retrieval test disrupted fear renewal (ABC paradigm). Second, behavioral training in fear renewal was accompanied by an increase in intracellular cAMP in the DG. When cAMP signaling in the DG was activated or disrupted, fear renewal was increased or impaired, respectively. As cAMP‐degrading phosphodiesterases, the protein levels of PDE4 isoforms PDE4A, PDE4A5, and PDE4D were increased in fear renewal in response to the change in cAMP levels. In addition, FSK and rolipram facilitated the acquisition of fear conditioning in long‐term memory, but not in short‐term memory, while Rp‐cAMP impaired long‐term memory. For extinction, FSK and rolipram inhibited extinction process, while Rp‐cAMP facilitated fear extinction. Here, we propose that the DG may be a critical hub for fear renewal and that cAMP signaling plays a major role in regulating fear renewal. Furthermore, fear retrieval and fear renewal may share cAMP signaling pathway in the DG.

So far, there is no direct evidence that synaptic plasticity of the DG is involved in renewal of conditional fear after extinction. However, the role of hippocampus in contextual memory retrieval has been shown in many previous studies. Inactivation of the ventral hippocampus (Hobin et al., 2006) and the dorsal hippocampus could disrupt renewal of conditional freezing to an extinguished tone CS outside of the extinction context (Corcoran & Maren, 2001). Consistent with this study, ventral hippocampal Fos expression was increased in fear renewal (ABC paradigm, auditory fear extinction) (Jin & Maren, 2015). Although cannula placements in these studies included the DG, they also contained adjacent areas, not targeting the DG. However, these data appear to contradict at least one recent study demonstrating a role for DG in the retrieval of contextual fear extinction (Bernier et al., 2017). It is speculated that this discrepancy is due to the DG contributes to contextual fear extinction retrieval or auditory fear extinction retrieval through a different mechanism.

In our study, inactivation of the DG resulted in the disruption of fear renewal, and the rats exhibited extinction memory. Previous studies revealed that extinction is a process that requires both the retrieval of hippocampally stored information and the acquisition of new learning that may be hippocampal‐dependent, at least in part (Bernier et al., 2017). The animals may require two memories for extinction retrieval: a fear conditioning memory and an extinction memory. In the hippocampus, there is a balance between a non‐DG‐dependent mechanism (direct perforant path‐CA1 inputs) and a DG‐dependent mechanism (indirect tri‐synaptic circuit DG‐CA3‐CA1 inputs). DG inhibition could cause a rapid loss of SC synaptic plasticity linking CA3 and CA1 and conditioned responding to CS (Madroňal et al., 2016). When the DG is inhibited before memory retrieval, potentiation via the indirect pathway is suppressed, unmasking depotentiation in the direct pathway and resulting in the expression of an extinction memory. In addition, another possible explanation for the role of the DG in fear renewal is that hippocampus cannot enhance the activity of “fear neurons” in the basal amygdala after inhibition of the DG, leading to the expression of an extinction memory (Orsini & Maren, 2012). Taken together, the present data suggest that the DG is critical for fear renewal.

cAMP has widely been demonstrated to play a key role in the cellular mechanisms underlying LTP and memory. AC1 is a neuron‐specific synaptic enzyme that contributes to Ca^2+^‐stimulated cAMP production (Zheng et al., 2016). LTP in AC1 knockout (AC1^−/−^) mice is significantly impaired, which is related to the decrease in cAMP concentration. Conversely, an increase in cAMP levels results from LTP induction in the CA1 (Otmakhova, Otmakhov, Mortenson, & Lisman, 2000). Furthermore, behavioral fear conditioning (CS paired with US) is due to a postsynaptic contribution of Ca^2+^ influx during the paired spike activity, which enhances the activity of AC (Kandel, 2012). Extinction is a process in which the repeated presentation of CS extinguishes the CS‐US association, resulting in a decreased conditioned fear response (Chen, Wang, Wang, & Li, 2017). If CS is presented outside of the conditioning context, the fear memory will be recovered. Therefore, the fact that synaptic efficiency is enhanced by fear conditioning and decreased by extinction and that a fear memory and an extinction memory must co‐exist in fear renewal may explain the relationship of cAMP levels in the groups of this study (the NE group > the ABC group > the ABB group).

In this study, inhibiting PKA by Rp‐cAMPs not only disrupted fear retrieval, but also impaired fear renewal. Inhibitors of PKA to hippocampal slices prevented the induction L‐LTP in the DG, that is to say, PKA may be necessary for the long‐term protein synthesis‐dependent changes that underlie L‐LTP (Schafe & Ledoux, 2000). In our study, fear retrieval tested 48 hr after fear conditioning which could be considered as a kind of long‐term fear memory. And fear renewal was reactivated two memories competition at test, the memory from acquisition and the memory from extinction, which could also be considered as long‐term memories (Laborda & Miller, 2012). Therefore, it is speculated that fear retrieval and fear renewal may share a common PKA molecular substrate.

Our study also demonstrated that PKA could act to acquisition of fear conditioning and extinction, consistent with previous studies (Isiegas et al., 2006). As above mentioned, PKA may be necessary for the long‐term fear memory, which can explain why long‐term memory could be affected, not short‐term memory. Furthermore, extinction can occur through a temporary weakening of the original memory, may be through a depression of the CS‐US association (Isiegas et al., 2006). Behaviorally, extinction and reconsolidation are functionally equivalent (Isiegas et al., 2006). Therefore, PKA may not only regulate original fear memory, but also extinction.

Likely to the effects of PKA activator Sp‐cAMP, rolipram could facilitate both fear retrieval and fear renewal. Such interference could be driven by elevation of cAMP levels and subsequent modulation of heightened PKA activity (Mueller, Hofmann, & Cherry, 2010) This interpretation is consistent with Barad, Bourtchouladze, Winder, and Kandel (1998), who reported that rolipram enhances retrieval of fear memory, as well as rolipram given prior to training also improve contextual fear conditioning in vivo, and long‐term potentiation in vitro is enhanced when hippocampal slices are stimulated in solution of rolipram. And there is also substantial evidence to suggest that rolipram can improve long‐term hippocampal‐dependent fear memory (Mueller et al., 2010). The DG has been verified a role in context fear context acquisition and fear extinction. And fear renewal is a memory involved hippocampal coordination of neuronal activity. Therefore, rolipram in the DG may play a role in fear renewal.

The protein level of PDE4A5 was significantly decreased in the ABC group compared to the ABB group. And it could see that the ABC group has a reversal tendency in decreasing the elevated level of the ABB group compared to the NE group. PDE4A5 regulates memory is by negatively impacting cAMP‐dependent signaling, which could explain our data of PDE4A5 expression in response to cAMP concentrations. A previous study reported that hippocampal PDE4A5 levels could impair long‐term memory (LTM) formation in contextual fear conditioning and FSK‐induced synaptic plasticity (Havekes et al., 2016). In contrast, in our study, it is speculated that fear renewal decreased PDE4A5 protein levels and then facilitated synaptic plasticity In the DG.

In our study, PDE4B expression did not show any significant differences among all experiment groups, while PDE4D was significantly decreased in the NE and ABC groups, similar to PDE4A5 expression. In the present study, extinction memory was tested 24 hr after extinction training, which assessed LTM. The different roles of PDE4B and PDE4D in fear renewal might be explained by their different functions in L‐LTP on cAMP signaling. It has been previously described that PDE4B^‐/‐^ mice display markedly enhanced long‐term depression (LTD) and unchanged LTP, as shown by electrophysiological recordings (Rutten et al., 2011), while PDE4D^‐/‐^ mice manifest enhanced LTP but unchanged LTD (Schaefer et al., 2012). On the behavioral level, PDE4B^‐/‐^ mice displayed impaired spatial reversal learning but without changes in fear conditioning, whereas PDE4D^‐/‐^ mice displayed obvious impairments in fear conditioning (Schaefer et al., 2012). It is possible that expression or involvement of PDE4B in the DG is not critical for L‐LTP and does not result in elevated cAMP in the DG. Thus, our findings suggest that different subtypes of PDE4 may regulate different pools of cAMP and support a role for compartmentalized cAMP signaling in regulating synaptic activity. Future studies might be focused on the different molecular and cellular mechanism of these PDE4 isoforms in fear renewal.

In a word, our data in this study suggested that cAMP signaling may have effects in fear renewal, although required for acquisition of fear memory and extinction. The basic demonstration in our experiments that the same signaling pathway impair original memory formation may facilitate extinction is consistent with many previous studies that initial learning and extinction shared similar molecular mechanism. The specific ways in which PKA may act on during fear conditioning and extinction remains to be determined. Understanding these mechanisms may help translate basic research to clinical settings, where extinction is a commonly used therapeutic intervention for psychiatric disorders involving fear (Bouton, Mineka, & Barlow, 2001; Milad & Quirk, 2002).

## CONFLICT OF INTERESTS

None.

## AUTHOR CONTRIBUTIONS

Xue‐Ling Ou and Xiao‐Guang Wang contributed to the conception of the work. Yan‐Wei Shi designed and collected the data; Bu‐Fang Fan collected the data; Li Xue analyzed the data; Yan‐Wei Shi wrote the article. All the authors discussed the results and commented on the manuscript. All the authors approved the final version of the manuscript.
